# Proteotoxic stress-induced apoptosis in cancer cells: understanding the susceptibility and enhancing the potency

**DOI:** 10.1038/s41420-022-01202-2

**Published:** 2022-10-04

**Authors:** Luca Iuliano, Emiliano Dalla, Raffaella Picco, Showmeya Mallavarapu, Martina Minisini, Eleonora Malavasi, Claudio Brancolini

**Affiliations:** Department of Medicine, Università degli Studi di Udine, P. le Kolbe 4 - 33100, Udine, Italy

**Keywords:** Targeted therapies, Apoptosis

## Abstract

Leiomyosarcoma (LMS) is aggressive cancer with few therapeutic options. LMS cells are more sensitive to proteotoxic stress compared to normal smooth muscle cells. We used small compound 2c to induce proteotoxic stress and compare the transcriptomic adaptations of immortalized human uterine smooth muscle cells (HUtSMC) and LMS cells SK-UT-1. We found that the expression of the heat shock proteins (HSPs) gene family is upregulated with higher efficiency in normal cells. In contrast, the upregulation of BH3-only proteins is higher in LMS cells. HSF1, the master regulator of HSP transcription, is sequestered into transcriptionally incompetent nuclear foci only in LMS cells, which explains the lower HSP upregulation. We also found that several compounds can enhance the cell death response to proteotoxic stress. Specifically, when low doses were used, an inhibitor of salt-inducible kinases (SIKs) and the inhibitor of IRE1α, a key element of the unfolded protein response (UPR), support proteotoxic-induced cell death with strength in LMS cells and without effects on the survival of normal cells. Overall, our data provide an explanation for the higher susceptibility of LMS cells to proteotoxic stress and suggest a potential option for co-treatment strategies.

## Introduction

Controlling the integrity and folding of proteins is a fundamental task of every cell and a crucial aspect of life and death options. A complex network of activities known as the proteostasis network (PN) serves to control protein homeostasis. Molecular chaperones and the ubiquitin–proteasome system (UPS) are the two main arms of the PN. When cells are confronted with conditions (proteotoxic stress) that lead to massive protein misfolding, they employ important adaptive responses [1, 2].

Transcription of chaperones and of UPS elements is strongly induced and translation is redirected in favor of those mRNAs that constitute the unfolding protein response (UPR). Phosphorylation of eIF2α (eukaryotic translation initiation factor 2α) leads to global attenuation of CAP-dependent translation and potentiation of selective translation of UPR mRNAs (CAP-independent translation) [3]. All cellular efforts are aimed at overcoming proteotoxic stress. If it is not overcome, cell death by apoptosis is a possible option [4, 5].

In several cancers, the overall level of proteotoxic stress is elevated due to the accumulation of point mutations, aneuploidy, and the increased rate of protein biosynthesis, an inherently error-prone process [4, 6, 7]. The potential susceptibility of cancer cells to proteotoxic stress has been exploited as a therapeutic option [7, 8]. To be efficient as a therapeutic approach, the induction of proteotoxic stress should have low toxicity in normal cells. We have recently shown that 2c, a small compound that induces proteotoxic stress, can antagonize tumor growth in vivo with lower overall toxicity by inhibiting deubiquitylases and altering protein folding [4, 9]. In this manuscript, we compared the adaptive response of normal and cancer cells to the induction of proteotoxic stress. We also investigated different co-treatments to find new agents that can enhance cell death of cancer cells in response to proteotoxic stress.

## Results

### Normal and cancer cells engage different transcriptomic adaptations in response to 2c-induced proteotoxic stress

**Fig. 1:**
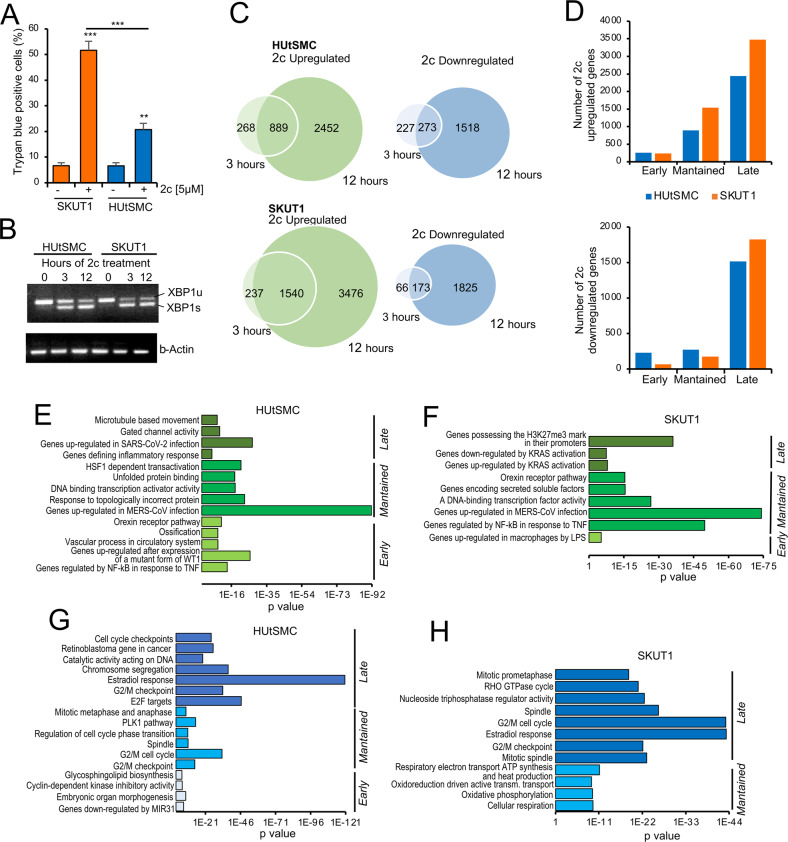
Transcriptomic adaptations in normal and cancer cells during proteotoxic stress. **A** Cell death in SK-UT-1 and HUtSMC after 24 h of incubation with 2c. Cell death was calculated as a percentage of cells positive to Trypan blue staining. **B** Agarose gel electrophoresis of RT-PCR products for the full-length *XBP1* transcript (*XBP1u*) and the spliced form (*XBP1s*). *β-Actin* was used as control. **C** Venn diagrams showing the number of transcripts upregulated and downregulated as indicated. 2c was used at 5 µmol/L concentration. **D** Total number of genes upregulated and downregulated in response to 2c-induced proteotoxic stress in HUtSMC and LMS cells only at 3 h (early genes) at both 3 and 12 ho (maintained genes) and only at 12 h (late genes). **E** Functional enrichments using the GSEA and the Molecular Signatures Database (MSigDB) tools. The analysis was performed for the indicated groups of upregulated genes in HUtSMC. Only the top terms are indicated. The detailed data are shown in Supplementary Table S1. **F** As in **E**, but the analysis was performed for the indicated groups of upregulated genes in SK-UT-1 cells. The detailed data are shown in Supplementary Table S1. **G** As in **E**, but the analysis was performed for the indicated groups of downregulated genes in HUtSMC. The detailed data are shown in Supplementary Table S2. **H** As in **E**, but the analysis was performed for the indicated groups of downregulated genes in SK-UT-1 cells. The detailed data are shown in Supplementary Table S2.

The small compound 2c makes covalent adducts with free thiols by Michael addition. In this way, it alters the activity of UPS, inhibiting deubiquitinylases, lowering glutathione levels, and inducing proteotoxic stress [10–12]. LMS are rare and aggressive soft tissue tumors that have smooth muscle cell characteristics. Surgical resection is an effective curative method for localized disease. Unfortunately, patients often relapse and available cytotoxic therapies show only modest clinical activity [13, 14]. We have recently shown that uterine LMS cells are sensitive to proteotoxic stress-induced apoptosis, whereas normal human uterine smooth muscle cells (HUtSMC) are more resistant [5]. To understand the molecular basis of this resistance, we monitored transcriptional adaptations to proteotoxic stress in cancer and normal cells. To avoid possible entry into replicative senescence a condition that may influence the level of proteotoxic stress [15], HUtSMC were immortalized with hTERT. Immortalization allows us to work with continuously cycling non-transformed cells and thus avoid the occurrence of senescence. Compared to LMS cells (SK-UT-1), immortalized HUtSMC-hTERT are partially resistant to cell death induced by 2c (Fig. 1A), like primary HUtSMC [5].

The transcription factor X-box binding protein 1 (XBP1) is a marker of ER stress and a key effector of the UPR. Activation of XBP1 occurs through non-conventional splicing [16]. To compare the induction of proteotoxic stress and activation of the UPR between HUtSMC and SK-UT-1, we measured the occurrence of XBP1 splicing using RT-PCR (Fig. 1B). At 3 h after 2c treatment, SK-UT-1 cells showed a slightly higher increase in XBP1 splicing compared with HUtSMC, but at 12 h, XBP1 splicing was comparable between the two cell lines (Supplementary Fig. S1A and B). We conclude that XBP1 splicing and UPR are induced with comparable intensity and kinetics in the two cell lines in response to 2c-induced proteotoxic stress.

For RNAseq experiments, two-time points were selected: 3 h after 2c treatment to assess the early response and 12 h, to gain insight into the later adaptive responses to proteotoxic stress. Principal component analysis (PCA) revealed the high reproducibility of the three biological replicates analyzed (Supplementary Fig. S2A). LMS cells upregulated a greater number of genes at both 3 and 12 h (Fig. 1C). In contrast, the number of downregulated genes was higher in normal cells at 3 h (500 versus 239) and in LMS cells at 12 h (Fig. 1C). Subsequently, DEGs were divided into three groups: the early group (regulated only after 3 h of treatment), the maintained group (regulated after both 3 and 12 h), and the late group (regulated only after 12 h) (Fig. 1C). 2c modulated the expression of a higher number of downregulated genes in non-transformed cells compared with cancer cells only in the early and maintained categories (Fig. 1D). In contrast, 2c upregulated a greater number of genes in SK-UT-1 cells within the maintained and late categories. LMS cells were also characterized by a higher number of late downregulated genes. In conclusion, non-transformed cells are more prone to downregulate genes in response to proteotoxic stress, whereas LMS cells were characterized by a higher number of upregulated genes at both time points examined.

The GSEA and the Molecular Signatures Database (MSigDB) were used to understand the cellular processes regulated by the different DEGs [17]. Figure 1E, F and Supplementary Table S1 show the top identified categories for the upregulated genes. In both normal and transformed cells, the early upregulated genes are related to the inflammatory response. In non-transformed cells, some differentiation pathways and the orexin receptor pathway are also upregulated. The orexin receptor pathway is also enriched among the maintained genes in LMS cells. Interestingly, a possible influence of this receptor system on ER stress was observed [18].

Unfolded protein response and HSF1 target genes are the top categories for the maintained genes in HUtSMC. They mark the cellular response to the induction of proteotoxic stress [1, 19]. Other enriched categories are transcription factors (TFs) and cellular response to MERS-CoV infection. These two categories are also found in SK-UT-1 cells. However, transformed cells upregulate many more TFs compared with HUtSMC (Supplementary Fig. S2B and Table S1). This fact may explain the higher number of upregulated genes in SK-UT-1 cells in response to 2c. Interestingly, the gene signature shared with MERS-CoV infection includes chaperones and inflammatory genes, among others (Supplemental Table S1), with several (*n* = 69) common between the two cell lines and 33 specific for HUtSMC and 38 for SK-UT-1 cells. This signature may indicate changes in PN induced by viral infection [20, 21]. Different categories characterize the late gene response. The response to SARS-Cov2 infection and inflammation is again enriched in HUtSMC, while in SK-UT-1 cells there are no clear definitions, as both RAS-induced and repressed genes are enriched.

In addition to inducing apoptosis, 2c-mediated proteotoxic stress leads to cell cycle arrest. As expected, the early response in HUtSMC is characterized by cell cycle-related genes among the downregulated genes (Fig. 1G, Supplemental Table S2). This is also true for the maintained and late response genes. Downregulated genes involved in the G2/M transition and mitotic activities are part of the maintained and late response. In SK-UT-1 cells, only the maintained and late downregulated genes are significantly enriched. Again, cell cycle-related and mitotic genes are the most enriched categories (Fig. 1H, Supplementary Table S2). A distinctive feature of SK-UT-1 cells is the downregulation of mitochondrial genes related to oxidative phosphorylation.

We also examined the genes common to the two cell lines, focusing on those of the maintained category (Supplementary Fig. S3A, B, Table S3). 459 genes were upregulated in common between the two cell lines, whereas only 40 genes were downregulated. Among the upregulated shared genes, the response to MERS-CoV infection was the most enriched category (Supplementary Fig. S3C).

### Differential regulation of BCL2 family member expression in non-transformed and cancer cells in response to 2c-induced proteotoxic stress

**Fig. 2:**
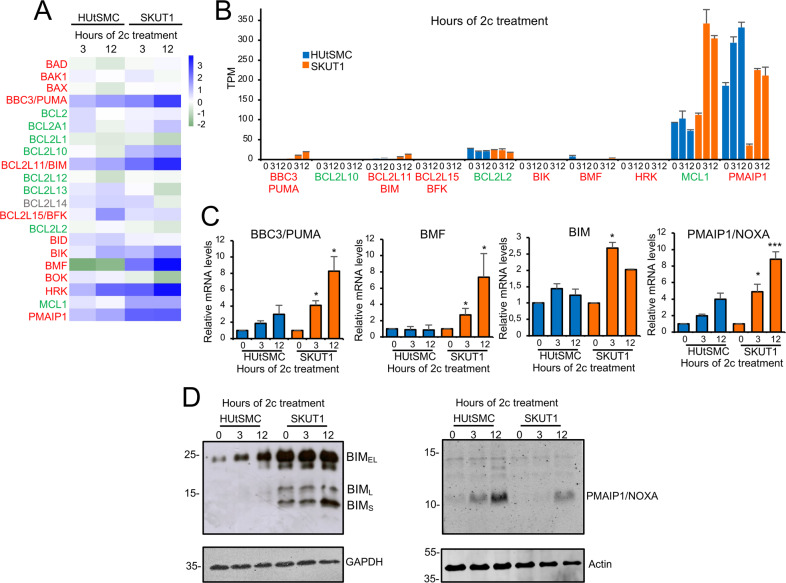
The expression of BCL2-family members in response to 2c-induced proteotoxic stress. **A** Heat-map reporting the expression levels (log2-fold change relative to untreated cells) of the BCL2 family members in response to 2c-induced proteotoxic stress in non-transformed and LMS cells. Anti-apoptotic members are indicated in green and pro-apoptotic in red. In gray BCL2L14 which activity is still debated. **B** Expression of the indicated BCL2-family members in HUtSMC and SK-UT-1 cells treated or not with 2c for the indicated times. Expression values are shown in transcripts per million (TPM) calculated from a gene model where isoforms were collapsed to a single gene. **C** qRT-PCR analysis of the mRNA levels of the indicated BH3-only members genes in HUtSMC or SK-UT-1 cells treated with 5 µmol/L of 2c for the indicated times. **D** Cells were treated for the indicated times with 2c (5 µmol/L). Immunoblots were performed using the indicated antibodies. Actin or GAPDH were used as the loading control.

HUtSMC are more resistant to proteotoxic-induced apoptosis compared with SK-UT-1 cells. In addition, SK-UT-1 shows changes in mitochondrial gene expression. BCL2 family members are key regulators of apoptosis. Through a complex network of interactions within the outer mitochondrial membrane, they control the efflux of mitochondrial killer proteins and the initiation of cell death [22]. It is possible that BCL2 family members are differentially regulated in response to proteotoxic stress in the two cell lines. Therefore, we compared the variations in the expression levels of all BCL2 family members between HUtSMC and SK-UT-1 cells in response to 2c treatment (Fig. 2A). The most important changes involved BH3-only proteins. *BBC3/PUMA, BCL2L11/BIM, BCL2L15/BFK, BIK, BMF, HRK*, and *PMAIP1*/*NOXA* are all upregulated in response to proteotoxic stress. Apart from *BFK*, the upregulation of all BH3-only proteins was much more consistent in cancer cells.

To confirm the effects of these upregulations, we compared the transcript per million (TPM) of 2c-upregulated BH3-only proteins in the two cell lines. We also examined *MCL1*, a member of the anti-apoptotic BCL2 family that is upregulated in response to proteotoxic stress and is critical for survival [23]. Except for *PMAIP1/NOXA*, the upregulated BH3-only are expressed at low levels (Fig. 2B). However, transcripts are more numerous in LMS cells. *PMAIP1/NOXA* again shows a peculiar behavior, being expressed at higher levels in normal cells and is more consistently upregulated in cancer cells. Differential upregulation of BBC3/PUMA, BMF, BIM, and PMAIP1/NOXA between non-transformed and cancer cells was confirmed by RT-PCR (Fig. 2C). However, analysis of TPM suggests that HUtSMC express higher levels of PMAIP1/NOXA. In addition, three different major isoforms of BIM (BIM_EL_, BIM_L_, and BIM_S_) are generated by alternative splicing. These isoforms exhibit different proapoptotic activities, with BIM_S_ being the most potent [24, 25]. Immunoblot analyses were performed to address these questions (Fig. 2D). Immunoblots confirmed the RNAseq data, with HUtSMC expressing higher levels of PMAIP1/NOXA. In contrast, LMS cells express higher levels of BIM proteins and specifically upregulate BIM_S_ in response to proteotoxic stress. In conclusion, the differential levels and induction of some BH3-only proteins in LMS cells may explain their higher apoptotic susceptibility to proteotoxic stress.

### Chaperone proteins are upregulated with higher efficiency in non-transformed cells

**Fig. 3:**
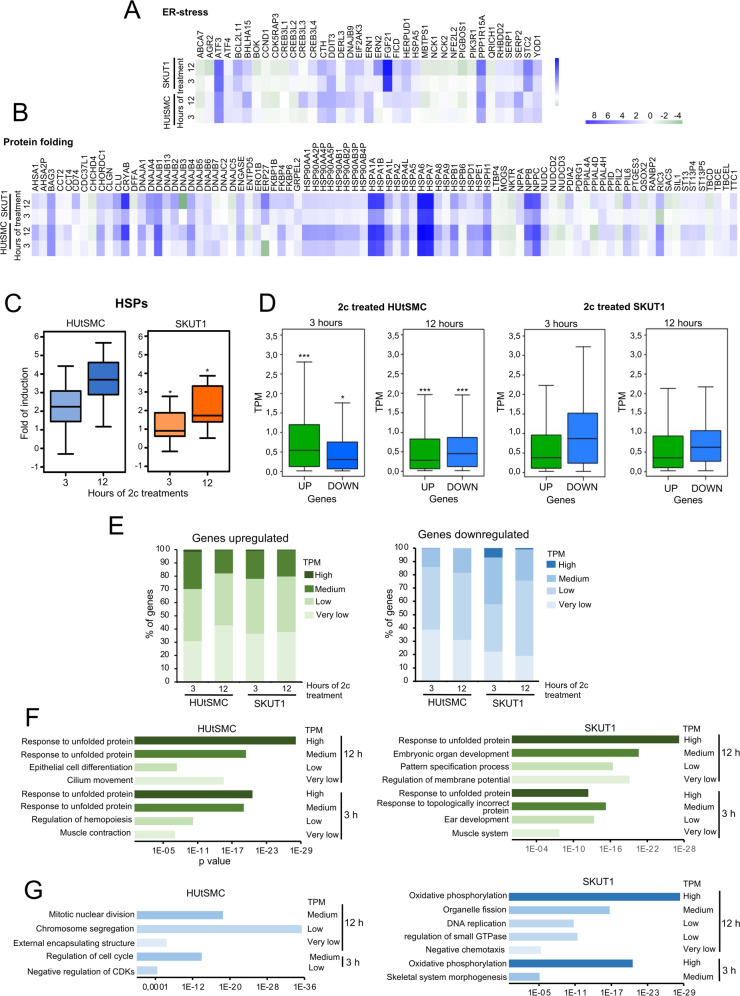
HSPs are upregulated at higher levels in non-transformed cells. **A** Heat-map reporting the expression levels (log2-fold change relative to untreated cells) of DEGs after 2c-induced proteotoxic stress and belonging to the UPR GO category in non-transformed and LMS cells. **B** As in **A** with genes belonging to the protein folding GO category in non-transformed and LMS cells. **C** Average median induction of HSPs differential regulated in response to 2c-induced proteotoxic stress (listed in **B**) in HUtSMC and SK-UT-1 cells. **D** TPM values are shown for DEGs in response to 2c-induced proteotoxic stress. TPM measure was calculated from a gene model where isoforms were collapsed into a single gene. Significances were tested using the Mann–Whitney *U* test. **E** Percentage of DEGs (up or downregulated) for the indicated TPM categories after 3 or 12 h from 2c treatment. **F** Functional enrichments using the GSEA and the Molecular Signatures Database (MSigDB) tools. The analysis was performed for the indicated groups of upregulated genes in HUtSMC and SK-UT-1 cells at the indicated time points. Only the top terms are indicated. The detailed data are shown in Supplementary Table S4. **G** As in **F**, but the analysis was performed for the indicated groups of downregulated genes in HUtSMC and SK-UT-1 cells at the indicated time-points. Only the top terms are indicated. The detailed data are shown in Supplementary Table S5.

The UPR in the endoplasmic reticulum (ER) and the cytoplasmic heat shock response (HSR) monitored by heat shock factor 1 (HSF1) are the two major responses triggered by proteotoxic stress to restore proteostasis [1, 4, 8, 26]. The differential regulation of BH3-only proteins described above might be related to different abilities to cope with proteotoxic stress. Therefore, we analyzed whether HUtSMC and SK-UT-1 cells exhibit peculiarities in the activation of the UPR and HSR. To investigate this aspect, we chose the GO categories “protein folding”, which includes 204 genes, and “endoplasmic reticulum unfolded” with 74 genes. Then, the expression level of these genes was analyzed in cells treated with 2c. Data are shown as a heat map only for the statistically significantly regulated genes (Fig. 3A, B). There was no clear differential expression pattern for the ER unfolded category (Fig. 3A). Some genes are more upregulated in SK-UT-1 (*ATF3, FGF21, STC2*), while others are more induced in HUtSMC (*BHLHA15, SERP2*). Instead, protein folding category analysis revealed that non-transformed cells upregulated the expression of several HSPs chaperones at higher levels, compared with SK-UT-1 cells and at both time points analyzed (Fig. 3B). This result was also confirmed by the quantitative analysis performed for all HSPs (Fig. 3C).

HSPs must be expressed at high concentrations in the presence of massive protein misfolding to act efficiently on their client proteins. Therefore, in addition to folding induction, it is important to investigate the absolute expression levels of HSPs between the two cell lines. First, we comprehensively analyzed the baseline expression of all DEGs. Figure 3C shows the result of such analysis. Overall, genes upregulated in HUtSMC cells show significantly higher TPM (transcripts per million) during the early response compared with SK-UT-1 cells. The reverse is true for downregulated genes, with SK-UT-1 cells silencing genes expressed at higher levels compared to non-transformed cells. Next, we decided to divide basal gene expression into four categories (very low, low, medium, and high expressed) as suggested by others (https://www.ebi.ac.uk). Figure 3D shows this analysis. Non-transformed cells upregulated a higher number of genes belonging to the medium and high TPM categories (respectively, mRNAs with 11–1000 and >1000 TPM) only after 3 h. SK-UT-1 cells show a similar response at both time points, while only after 3 h do they upregulate a higher percentage of very low or low expressed genes (mRNAs with a concentration <0.5 and between 0.5 and 10 TPM), respectively. As shown in Fig. 3C, cancer cells downregulate a higher percentage of medium and high-expressed genes than normal cells, especially during the early response.

Finally, to clarify which cellular responses are monitored by the most abundant mRNAs, a GSEA was performed. The same categories identified in the previous analysis were confirmed when the up or downregulated genes were clustered for the TPM score (Fig. 3E, F). Importantly, the genes upregulated at 3 and 12 h after treatment belonging to the “response to unfolded proteins” category, a category that includes HSP genes, are characterized by numerous mRNA copies and are categorized as “medium” and “high” TPM in both cell lines. Because HSPs are most upregulated in non-transformed cells (Fig. 3B), they may provide greater efficiency in maintaining proteostasis. This differential adaptive response may account for the observed apoptotic resistance. Finally, genes controlling oxidative phosphorylation are abundantly expressed and specifically downregulated in cancer cells.

### Differential nuclear compartmentalization of HSF1 during 2c-induced proteotoxic stress between non-transformed and cancer cells

**Fig. 4:**
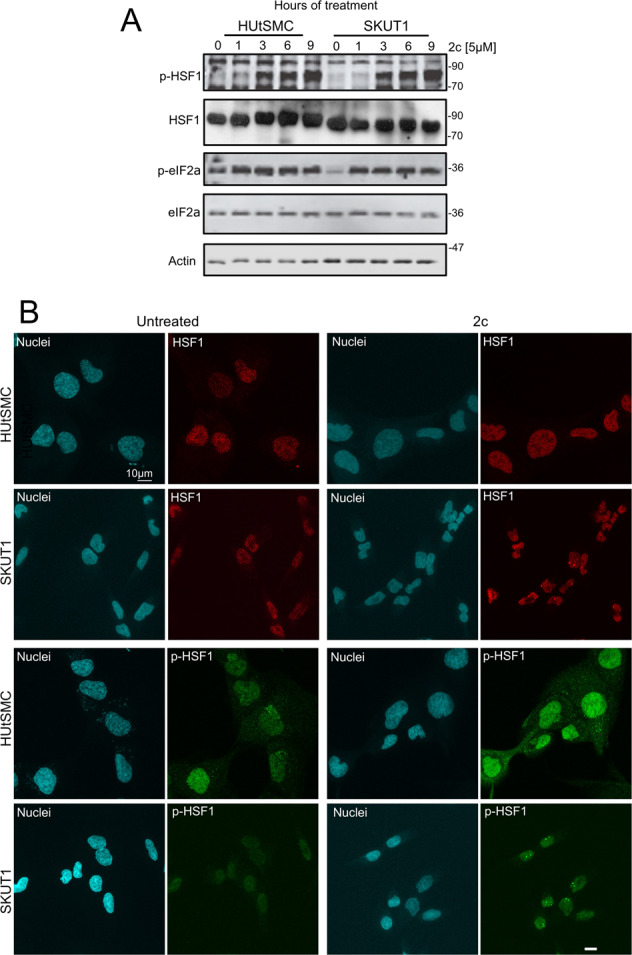
Regulation of HSF1 during 2c-induced proteotoxic stress between non-transformed and cancer cells. **A** Cells were treated for the indicated times with 2c (5 µmol/L). Immunoblots were performed using the indicated antibodies. Actin was used as a loading control. **B** Cells were treated or not with 5 µmol/L of 2c for 3 h. Immunofluorescence analysis was performed to visualize HSF1 and HSF1 phosphorylated at Ser 326. Nuclei were stained with Hoechst 33342. Confocal images are shown in pseudocolors. Bar 10 µm.

Heat shock transcription factor 1 (HSF1) is the master regulator of the heat shock response and upregulates the transcription of several HSP family chaperones [19]. The differential upregulation of HSPs between non-transformed and cancer cells might depend on the different engagements of HSF1. The activation of HSF1 after proteotoxic stress is controlled by different phosphorylation events [1, 26]. We monitored HSF1 activation by phosphorylation of Ser 326 [1, 19, 27]. Figure 4A shows that HSF1 is strongly phosphorylated at Ser 326 3 h after 2c treatment, with kinetics and intensity consistent between normal and cancer cells. We also analyzed the induction of ER-stress and the UPR by monitoring eIF2α phosphorylation, a PERK substrate. Compared with HSF1-Ser 326, eIF2α phosphorylation occurs much earlier, within 1 h of treatment, and remains constant throughout the analysis. Again, the pattern of UPR activation is similar between non-transformed and LMS cells.

During stress, HSF1 can accumulate in nuclear stress bodies called foci. Within these foci, transcriptional upregulation of chaperones by HSF1 is reduced, and cells eventually die by apoptosis [28]. Therefore, we used confocal microscopy to examine the appearance of HSF1-positive nuclear foci three hours after 2c-induced proteotoxic stress, when HSF1 phosphorylation is clearly visible. Importantly, these foci can only be detected in SK-UT-1 cells in more than 50% of cells. Often 2 foci/cells can be detected but in some cases more. In these nuclear foci, HSF1 is phosphorylated at Ser 326 (Fig. 4B).

Therefore, the difference in subnuclear compartmentalization of HSF1 may account for the differential upregulation of HSP family chaperones between non-transformed smooth muscle cells and LMS cells.

### Co-treatment screening to sensitize cancer cells to proteotoxic stress-induced cell death

The possibility of enhancing the apoptotic response to proteotoxic stress has been sporadically investigated in the past [29, 30]. To clarify this point, we investigated different co-treatments for their ability to sensitize LMS cells to apoptosis induced by proteotoxic stress. The different co-treatments were grouped according to their mechanism of action and include: (i) genotoxic agents used in LMS therapy (doxorubicin and gemcitabine), (ii) inhibitors of signaling kinases (Akt inhibitor MK2206; MAPK7/ERK5 inhibitor XMD8-92; mTOR inhibitor Torin1; salt inducible kinases (SIKs) inhibitor YKL-06-061 and MEK1/2 inhibitor Selumetinib), (iii) HDAC inhibitors SAHA, TMP195, and NKL54, (iv) autophagy inhibitors, bafilomycin A1 and chloroquine, (v) BCL2 inhibitors ABT199 and ABT263, and (vi) the IRE1α inhibitor MKC3946, which affects UPR (Supplementary Table S6). The concentration of each compound was chosen according to the references (Supplementary Table S6). The proteotoxic inducer 2c was used at a concentration of 2.5 µM.

**Fig. 5:**
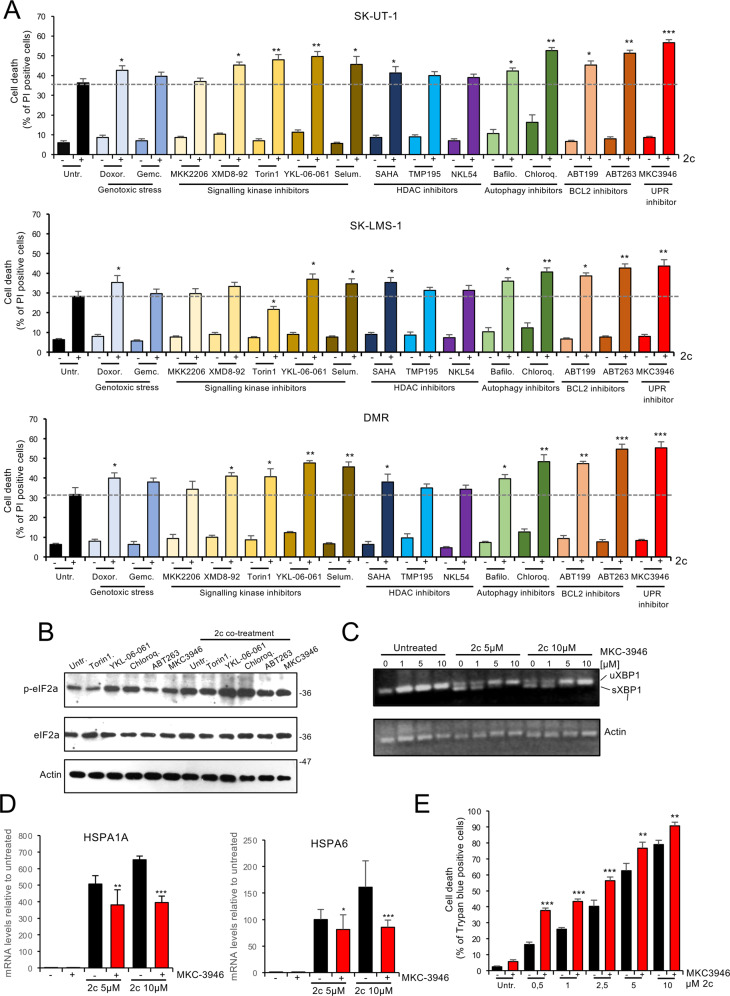
Identification of new compounds that potentiate proteotoxic-stress-induced cell death. **A** Cells were treated for 24 h with 2c (2.5 µmol/L), Doxorubicin (25 nmol/L), Gemcitabine (10 nmol/L), MK2206 (10 μmol/L), XMD8-92 (1 μmol/L), Torin1 (100 nmol/L), YKL-06-061 (1 μmol/L), Selumetinib (1 μmol/L), SAHA (2.5 μmol/L), TMP195 (20 μmol/L), NKL54 (5 μmol/L), Bafilomycin A1 (1 μmol/L), Chloroquine (1 μmol/L), ABT199 (100 nmol/L), ABT263 (100 nmol/L), MKC3946 (10 μmol/L), as indicated. **B** SK-UT-1 cells were treated for 4 h with 2c (2.5 µmol/L) Torin1 (100 nmol/L), YKL-06-061 (1 μmol/L), Chloroquine (1 μmol/L), ABT263 (100 nmol/L), or MKC3946 (10 μmol/L), as indicated. Immunoblots were performed using the indicated antibodies. Actin was used as a loading control. **C** Agarose gel electrophoresis of RT-PCR products for the full-length *XBP1* transcript (*XBP1u*) and the spliced form (*XBP1s*). Samples were from SK-UT-1 treated with 2c (5 or 10 μmol/L) alone or in combination with MKC3946 (10 μmol/L) for 4 h. *β-Actin* was used as control. **D** mRNA levels expression of *HSPA1A* and *HSPA6* in SK-UT-1 cells treated with 2c (5 or 10 μmol/L) alone or in combination with MKC3946 (10 μmol/L) for 4 h. **E** Cell death in SK-UT-1 cells after 24 h of incubation with the indicated concentrations of 2c in the presence or not of MKC3946 (10 μmol/L).

The screening was performed on three different LMS cell lines in parallel (SK-UT-1, SK-LMS-1, and DMR). Several compounds can sensitize cancer cells to apoptosis induced by proteotoxic stress. Except for the mTOR inhibitor, which is not active in SK-LMS-1 cells, the different compounds have similar effects on the different LMS cells tested. The strongest effects were obtained with the SIKs inhibitor YKL-06-061, the BCL2 inhibitor ABT263, and the IRE1α inhibitor MKC3946 (Fig. 5A). Activation of the UPR, as measured by eIF2α phosphorylation, was particularly enhanced by concomitant treatment with SIKs inhibitors or autophagy inhibitors, which could activate the UPR even in the absence of 2c (Fig. 5B).

We tested the efficacy of the IRE1α inhibitor in our model system. MKC3946 at a concentration of 10 µM was necessary to completely suppress XBP splicing in response to 2c-induced proteotoxic stress (Fig. 5C). Importantly, MKC3946 impaired the upregulation of HSPs expression, a possible mechanism that could explain the sensitization effect (Fig. 5D). A dose-dependent study was performed to evaluate, more comprehensively, the additive effect of MKC3946 on 2c-induced apoptosis. Blocking IRE1α was particularly effective when low concentrations of 2c were used (Fig. 5E). This observation suggests that the UPR is no longer able to counteract apoptosis once a high level of proteotoxic stress has been induced.

### Proteotoxic stress at low doses in the presence of the IRE1α inhibitor is manageable in non-transformed cells

**Fig. 6:**
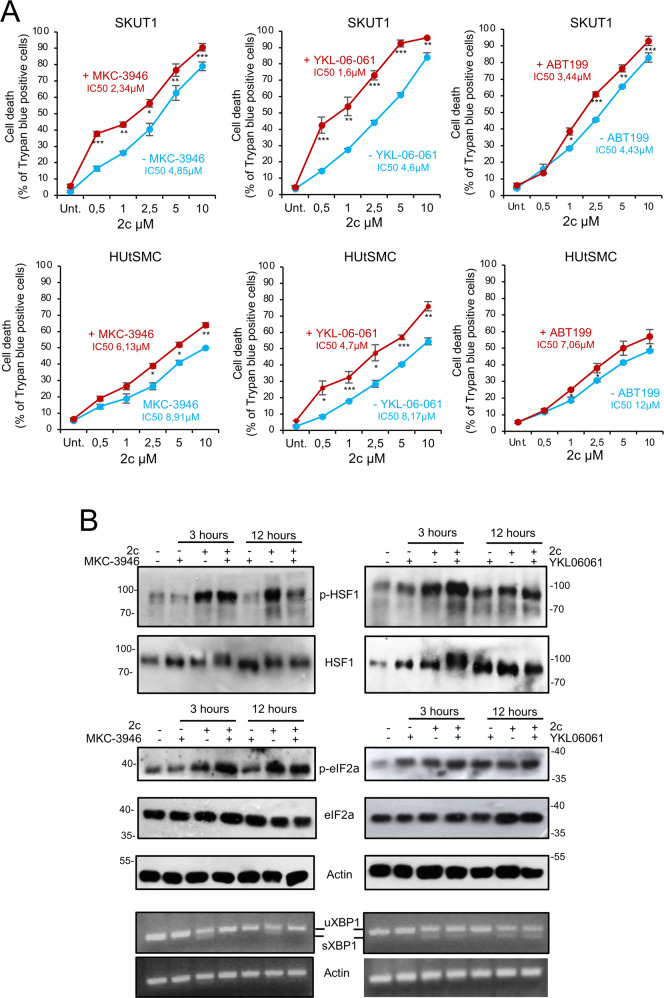
Effect of PERK and SIKs inhibitors on cell death in response to proteotoxic stress. **A** Cell death after 24 h of combined treatment with the indicated concentrations of 2c and MKC3946 (10 μmol/L), YKL-06-061 (1 μmol/L), and ABT199 (100 nmol/L). **B** SK-UT-1 cells were treated for 3 or 12 h with 2c (0.5 µmol/L) in the presence or not of MKC3946 (10 μmol/L) or of YKL-06-061 (1 μmol/L). Immunoblots were performed using the indicated antibodies. Actin was used as a loading control. RT-PCR products for the full-length *XBP1* transcript (*XBP1u*) and the spliced form (*XBP1s*) are also shown.

To be of interest from a clinical perspective, a combined treatment must have lower toxicity in normal cells compared with cancer cells. Therefore, dose-dependent studies were performed in SK-UT-1 and HUtSMC cells to understand whether SIKs and IRE1α inhibitors, which are among the most potent compounds identified in the screening, have lower toxicity in normal cells. For comparison, we also examined the effect of the BCL2 inhibitor ABT199, a less potent sensitizer of apoptosis in response to proteotoxic stress. SIKs and IRE1α inhibitors enhanced the apoptotic response to all concentrations of 2c tested in both normal and cancer cells. However, when low doses of 2c were used, their effect was much more pronounced in cancer cells than in normal cells (Fig. 6A). ABT199 confirmed its lower ability to increase apoptosis in response to 2c and is quite ineffective at low doses of 2c.

Finally, we examined the activation of HSF1 and the UPR in cells treated with low doses of 2c in the presence of IRE1α and SIKs inhibitors (Fig. 6B). Inhibition of IRE1α did not induce HSF1 and eIF2α phosphorylation. Low concentrations of 2c (0.5 µM) are sufficient to activate HSF1 and the UPR. Interestingly, HSF1 phosphorylation is reduced after 12 h of treatment in the presence of the IRE1α inhibitor, suggesting an effect of co-treatment on HSPs expression and cell survival. eIF2α activation is slightly increased in co-treated cells, but only after 3 h. In contrast, inhibition of SIKs activates the UPR, and an additive effect can be detected at 3 h in 2c co-treated cells.

## Discussion

The induction of proteotoxic stress is a relatively new therapeutic strategy that has attracted considerable interest [5, 7, 8, 30–32]. In this manuscript, we addressed two important issues: (i) understanding the reasons for the differential susceptibility of normal cells to proteotoxic stress compared to cancer cells and (ii) the possibility of enhancing the cell death response triggered by proteotoxic stress.

Transcriptomic adaptations to proteotoxic stress show differences between cancer and normal cells. Initially, cancer cells upregulate a greater number of genes, whereas in normal cells more genes are downregulated during the early response. Although translational control is an important aspect of the response to proteotoxic stress and a certain proportion of downregulated genes are highly expressed in cancer cells, we speculate that the requirements for new protein synthesis may be less demanding in normal cells, facilitating success in proteotoxic stress. In general, the top gene categories and gene functions modulated by proteotoxic stress are similar in cancer and normal cells. A distinctive feature of cancer cells is the downregulation of genes involved in oxidative phosphorylation. An effect that could be related to the impairment of mitochondrial functions due to the apoptotic response. Instead, the most striking difference was quantitative and involved the upregulation of several members of the HSPs family [1, 26, 33]. Normal cells upregulate the expression of several HSPs with higher efficiency than cancer cells. We, therefore, hypothesize that normal cells may acquire a higher ability to counteract protein misfolding, cope with proteotoxic stress, and thus escape cell death. This higher efficiency correlates with the different subcellular localization of HSF1, the master regulator of HSP transcription [1, 26, 28, 33].

HSF1 can accumulate in nuclear stress bodies called foci [28, 34]. We observed that HSF1 foci can be detected in LMS cells within 3 h of 2c-induced proteotoxic stress, whereas they were absent in non-transformed cells. Within these foci, HSF1 is unable to drive HSP transcription and cells eventually enter apoptosis [28]. This scenario is confirmed by our study. Because cancer cells might have a different propensity to form HSF1 foci [35], this observation needs to be validated in other models.

Lower upregulation of HSPs in SK-UT-1 cells correlates with increased upregulation of pro-apoptotic BH3-only proteins [22]. This differential upregulation of pro-apoptotic genes between normal and cancer cells may explain the increased survival of HUtSMC. *BBC3/PUMA, BMF, BIM*, and *PMAIP1/NOXA* are all more upregulated in cancer cells. Moreover, TPM for *BIM* and *BBC3/PUMA* are higher in SK-UT-1 cells. Therefore, not only the increase in expression but also the total mRNA level is higher in cancer cells.

*BIM, PMAIP1/NOXA*, and *BBC3/PUMA* transcription is subject to the control of various signaling pathways [4]. *PMAIP1/NOXA* and *BBC3/PUMA* can be transcribed by p53 but also by other pathways [36–38]. In response to protein misfolding and ER stress, the ATF4-CHOP axis (C/EBP homologous protein), which is a key arm of the UPR, controls the transcription of *BBC3/PUMA, PMAIP1/NOXA*, and *BIM* [4, 36–39]. *BMF* expression has not previously been associated with proteotoxic stress. However, since FOXO3 (Forkhead box transcription factor-3A) controls both *BMF* and *BIM* transcription [40, 41] and FOXO3 is under the control of UPS [42], the involvement of this axis in the induction of apoptosis by proteotoxic stress is highly plausible.

The possibility of improving the therapeutic value of proteotoxic stress by combined treatments is still an under-researched option [7, 8, 43]. We have identified several treatments that can enhance apoptosis in response to 2c-induced proteotoxic stress. MKC-3946, the inhibitor of (IRE1α) and of XBP1 splicing, can significantly enhance apoptosis in response to 2c-induced proteotoxic stress in all LMS cells tested. Blocking part of the UPR makes cells more susceptible to apoptosis. A similar effect of MKC-3946 was observed in multiple myeloma cells treated with bortezomib or the HSP inhibitor 17-AAG [44]. The SIK1/2/3 inhibitor YKL-06-061 also robustly enhanced apoptosis in response to proteotoxic stress. Interestingly, blocking SIKs activity is sufficient to increase eIF2α phosphorylation, suggesting ER stress induction. Thus, in contrast to the IRE1α inhibitor, the SIKs inhibitor acts by increasing proteotoxic stress. Although there is limited evidence for the role of SIKs in proteotoxic stress, the influence of SIK2 on ER-associated protein degradation (ERAD) [45] may explain the accumulation of misfolded proteins and the induction of ER stress.

In summary, we have highlighted possible explanations for the increased susceptibility of cancer cells to proteotoxic stress-induced apoptosis and identified co-treatments that could increase its selectivity. Although we have focused our studies on leiomyosarcoma cells, we are confident that these discoveries may have broad application, as similar responses may be triggered in other cancers.

## Materials and methods

### Cell culture conditions, drug treatments and cell death assay

LMS cell lines SK-UT-1, DMR, and SK-LMS-1 were validated by RNA profiling and grown as previously described [5]. The primary human uterine smooth muscle cells (HUtSMC) obtained from ATCC, were immortalized after lentiviral infection with hTERT. All cell lines were weekly tested to be free from *Mycoplasma* contamination using Hoechst 33342 (Sigma) staining and microscopic inspection. After drug treatment, cell death was assessed by using 10 μg/ml of Propidium Iodide (PI; Sigma) or with 0.1% Trypan Blue (TB; Sigma) staining. PI fluorescence positivity and cell positivity to TB were determined with Countess II FL automated cell counter (Invitrogen).

### Chemicals

The following chemicals were used: 2c [9], dimethyl sulfoxide (DMSO), Doxorubicin, MK2206, Torin1, Bafilomycin A1, Chloroquine, ABT263 (Sigma), Gemcitabine, XMD8-92, YKL-06-061, ABT199 (CSN Scientific), Selumetinib, TMP195, MKC3946 (MedChemExpress), SAHA (Cayman Chemicals), NKL54 [46].

### Antibodies and immunoblotting

Immunoblotting was performed as previously described [4, 47]. Primary antibodies were: β-Actin (13E5 #4970), eIF2α (#9722), p-eIF2α (Ser51; #9721), HSF1 (#4356), BIM (#2933), (Cell Signaling Technology), NOXA/PMAIP1 (Merck), GAPDH (ThermoFisher AM4300) and p-HSF1 (Ser326; #ab76076) (Abcam). After washes, membranes were incubated or with the horseradish peroxidase-conjugated secondary antibody (Sigma) and developed using Super Signal West Dura (Pierce Waltham) on a photographic film or with goat anti-rabbit IgG star bright blue 700 secondary antibodies (BioRad) by detecting the fluorescence using OdisseyCLx (LI-COR).

### RNA extraction, quantitative qRT-PCR and analysis of *XBP1* mRNA splicing

Cells were lysed using Tri-Reagent (Molecular Research Center). 1.0 μg of total RNA was retro-transcribed by using 100 units of M-MLV Reverse transcriptase (Life Technologies). qRT-PCRs were performed using SYBR green technology (KAPA Biosystems). Data were analysed by comparative threshold cycle using *HPRT* and *GAPDH* as normalizer. For the evaluation of XBP1 splicing, 1.5 μg of total RNA was retro-transcribed and the resulting cDNAs were amplified by PCR. The list of primers is in the Supplementary data.

### Immunofluorescence confocal microscopy

Immunofluorescence was done as previously described [6]. The secondary antibody used was anti-rabbit Alexa flour 488 (Thermo Fisher Scientific). Hoechst 33342 was used to counterstain nuclei. Images were acquired using a Leica TCS SP8 confocal microscope (Leica Microsystems). The contrast was linearly adjusted on the whole image.

### RNA-seq analysis

RNA was extracted with Quick-RNA MagBead kit (Zymo Research) in the presence of DNAse I. cDNA libraries were prepared from poly(A) selected RNA following Illumina specifications. Quality control for raw sequencing reads was performed using FastQC (v0.11.7) as previously described [46] (www.bioinformatics.babraham.ac.uk/projects/fastqc/) and the ShortRead R package (v1.50.0) [48]. Raw reads were clipped using the Trimmomatic software (v0.35) [49]. Reads with an average Phred quality lower than 28 were discarded. Reads were mapped to the Human reference genome downloaded from Ensembl (version 104) using STAR (v2.5.0) [50]. Transcript assembly and quantification were done with StringTie (v2.1.5) [51]. Differentially expressed genes (DEGs) were identified using the DESeq2 R package (v1.32.0) and applying the log2 fold change (|log2 FC|) > 1 and FDR < 0.05 thresholds (10). The functional enrichment analysis was performed using the ClusterProfiler (v4.0.5) R package querying gene set collections (msigdb.v7.5.1.symbols.gmt) downloaded from the Molecular Signatures Database [17]. Results with a Benjamini–Hochberg FDR-adjusted *p*-value < 0.05 were considered statistically significant. Heatmaps were generated with the pheatmap R package (v1.0.12) [52].

### Statistics

For experimental data Student's *t*-test was employed. For comparisons between samples >2, the ANOVA test was applied coupled to Kruskal–Wallis and Dunn’s Multiple Comparison Test. Statistical significance in data distributions was tested using the Wilcoxon test. We marked with *p < 0.05, **p < 0.01, ***p < 0.001. Unless otherwise indicated, all the data in the figures were represented as arithmetic means ± the standard deviations from at least three independent biological replicates.

## Supplementary information

Authorship change agreement

Supplemental material

Table S1

Table S2

Table S3

Table S4

Table S5

Table S6

Original Data File

## Data Availability

The data generated in this study are available as ‘private access token’ in Gene Expression Omnibus (GEO) at https://www.ncbi.nlm.nih.gov/geo/query/acc.cgi?acc=GSE205596.
