# Supplementary material for: Proteotoxic stress-induced apoptosis in cancer cells: understanding the susceptibility and enhancing the potency
Source: Cell Death Discov. 2022 Oct 4;8:407. doi: 10.1038/s41420-022-01202-2 (PMC9531228; doi:10.1038/s41420-022-01202-2)
Supplement: Supplementary file 2 — Supplemental material [file 41420_2022_1202_MOESM2_ESM.pdf]

Luca Iuliano[1#], Emiliano Dalla[1#], Raffaella Picco[1#], Showmeya Mallavarapu[1#], Martina Minisini[1], and Claudio Brancolini[1,2]

[1]Department of Medicine, Università degli Studi di Udine. P. le Kolbe 4 - 33100 Udine Italy.

[#]L. Iuliano, E. Dalla, R. Picco and S. Mallavarapu contributed equally for this article

[2]Address correspondence to: Claudio Brancolini claudio.brancolini@uniud.it

Supplementary data

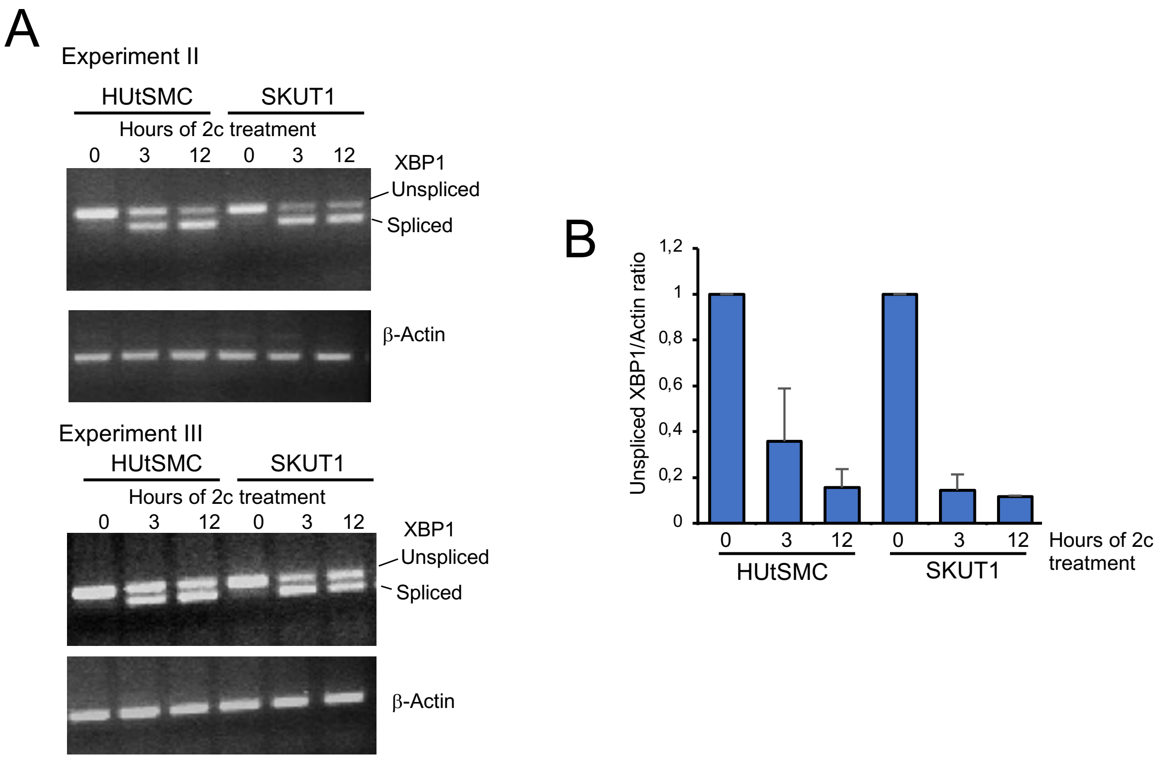

Figure S1. Activation of the UPR in response to 2c-induced proteotoxic stress.

A) Agarose gel electrophoresis of RT-PCR products for the full-length *XBP1* transcript (*XBP1u*) and the spliced form (*XBP1s*). *α-Actin* was used as control. Cells were incubated with 5µmol/L 2c for the indicated times.

B) Quantitative densitometric analysis of XBP1 splicing in response to the 2c treatment. Data are from 3 independent biological replicates. Cells were incubated with 5µmol/L 2c for the indicated times.

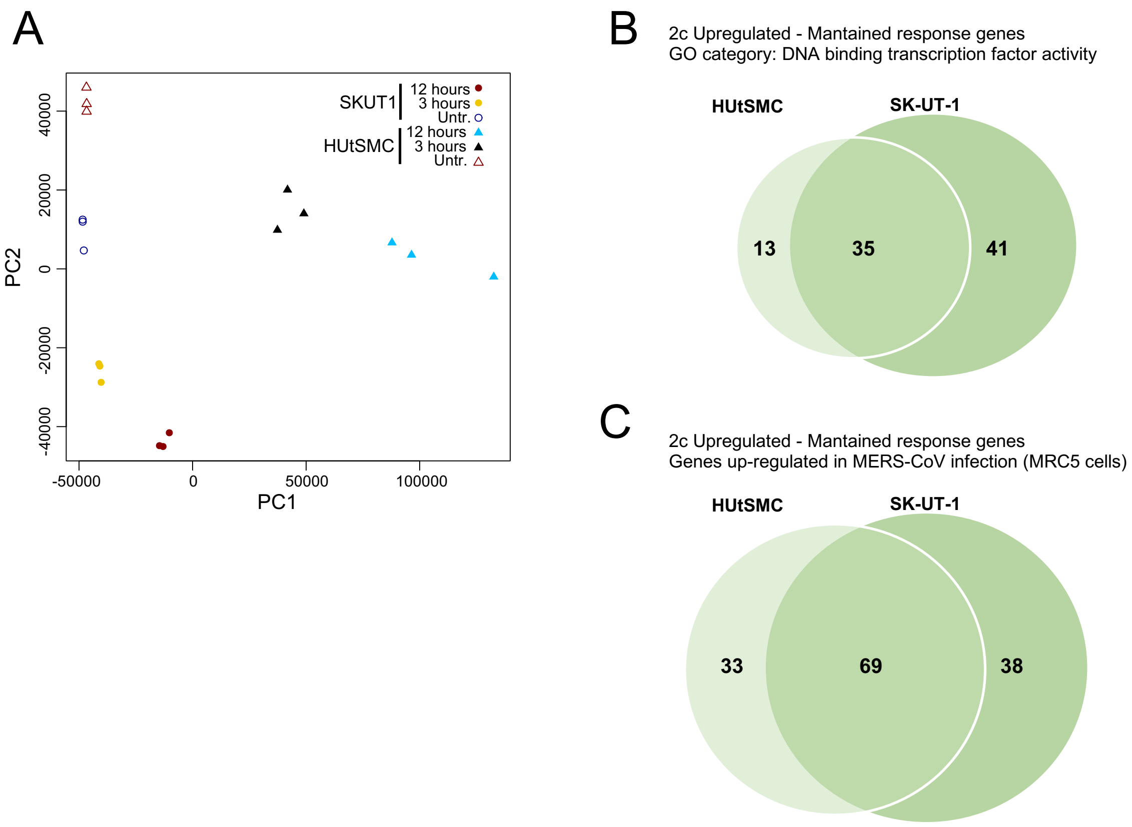

Figure S2. Comparative transcriptomic analysis in response to 2c-induced proteotoxic stress.

A) PCA analysis performed on the expression profiles of the indicated treatments and times in HUtSMC and SK-UT-1 cells.

B) Venn diagram showing the number of transcription factors (GO category) commonly and differentially upregulated in response to 2c-induced proteotoxic stress in HUtSMC and SK-UT-1 cells at 3 and 12 hours.

C) Venn diagram showing the number of genes upregulated during MERS-CoV infection in MRC5 cells (GSEA/MSigDB, Hallmark) commonly and differentially upregulated in response to 2c-induced proteotoxic stress in HUtSMC and SK-UT-1 cells at 3 and 12 hours.

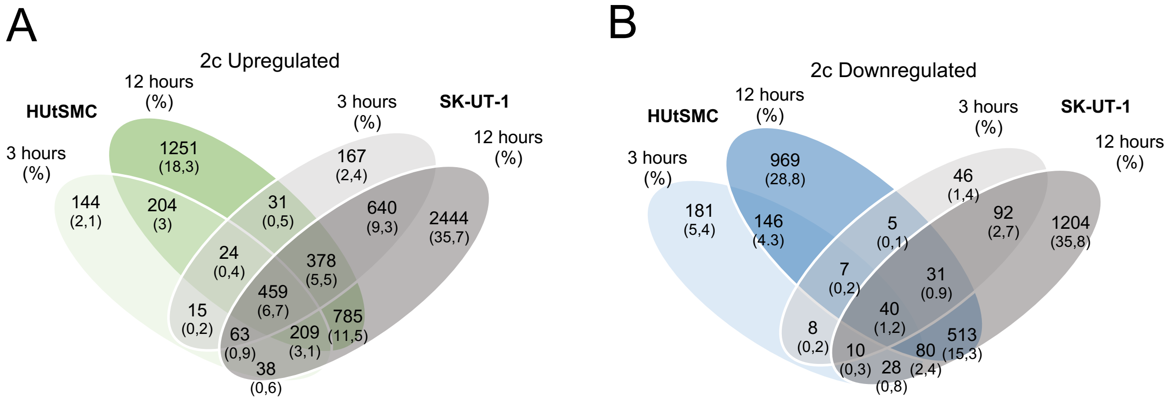

C

MAINTAINED COMMON RESPONSE			
ID	Description	pvalue	GeneRatio (%)
GOBP	Response to topologically incorrect protein	3.39E-20	15,19
Canonical pathways WP	HSF1-dependent transactivation	7,31E-15	31,58
Reactome	Orexin receptor pathway	1,91E-13	11,69
Chemical and genetic perturbations	Genes up-regulated in MERS-CoV infection	6,08E-70	23,31
Chemical and genetic perturbations	Genes up-regulated in MCF7 cells after stimulation with NRG1	1,33E-49	26,59
Hallmark gene set	Genes regulated by NF-kB in response to TNF	1,22E-40	21,0
Hallmark gene set	Genes involved in p53 pathways and networks.	2,37E-22	14
Hallmark gene set	Genes mediating programmed cell death (apoptosis) by activation of caspases	3,39E-14	11,8
Hallmark gene set	Genes up-regulated in response to low oxygen levels (hypoxia)	1,75E-12	9,5

Figure S3. Common and distinct transcriptomic adaptations in HUtSMC and SK-UT-1 cells during 2c-induced proteotoxic stress.

A) Venn diagram showing the number of transcripts commonly and differentially upregulated between HUtSMC and SK-UT-1 cells in response to 2c at the indicated hours.

B) Venn diagram showing the number of transcripts commonly and differentially downregulated between HUtSMC and SK-UT-1 cells in response to 2c at the indicated hours.

C) List of the most significantly enriched functional terms for the upregulated maintained genes common to the HUtSMC and SK-UT-1 cells.

Table S1. Functional enrichments using the GSEA and the Molecular Signatures Database (MSigDB) tools. The analysis was performed for the indicated groups of upregulated genes in HUtSMC and SK-UT-1 cells.

Table S2. Functional enrichments using the GSEA and the Molecular Signatures Database (MSigDB) tools. The analysis was performed for the indicated groups of downregulated genes in HUtSMC and SK-UT-1 cells.

Table S3. Functional enrichments using the GSEA and the Molecular Signatures Database (MSigDB) tools. The analysis was performed for the indicated groups of common and specific upregulated genes.

Table S4. Functional enrichments using the GSEA and the Molecular Signatures Database (MSigDB) tools. The analysis was performed for the indicated groups of upregulated genes at 3 or 12-hours from the 2c treatment in HUtSMC and SK-UT-1 cells, subdivided based on the selected TPM categories.

Table S5. Functional enrichments using the GSEA and the Molecular Signatures Database (MSigDB) tools. The analysis was performed for the indicated groups of downregulated genes at 3- or 12-hours from the 2c treatment in HUtSMC and SK-UT-1 cells, subdivided based on the selected TPM categories.

Table S6. Compounds used for the co-treatments
