# Supplementary material for: Proteotoxic stress-induced apoptosis in cancer cells: understanding the susceptibility and enhancing the potency
Source: Cell Death Discov. 2022 Oct 4;8:407. doi: 10.1038/s41420-022-01202-2 (PMC9531228; doi:10.1038/s41420-022-01202-2)

Original data figure 2D

NOXA

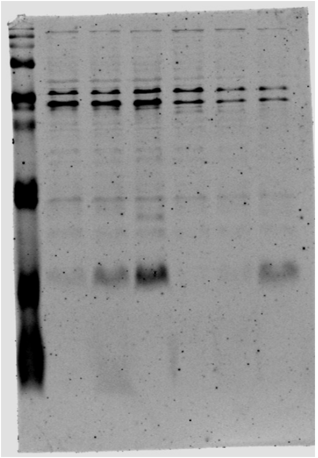

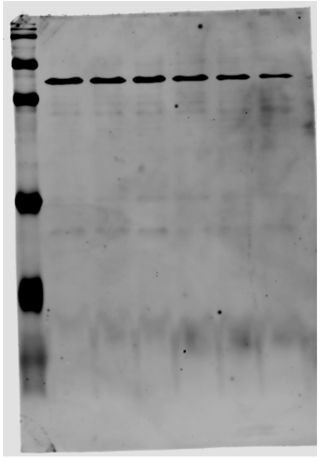

ACTIN

BIM

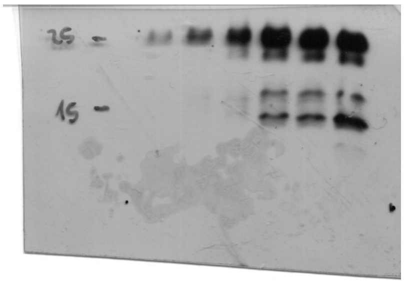

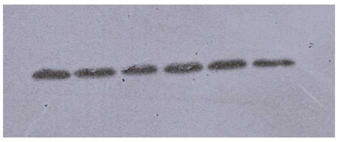

GAPDH

Original data figure 4A

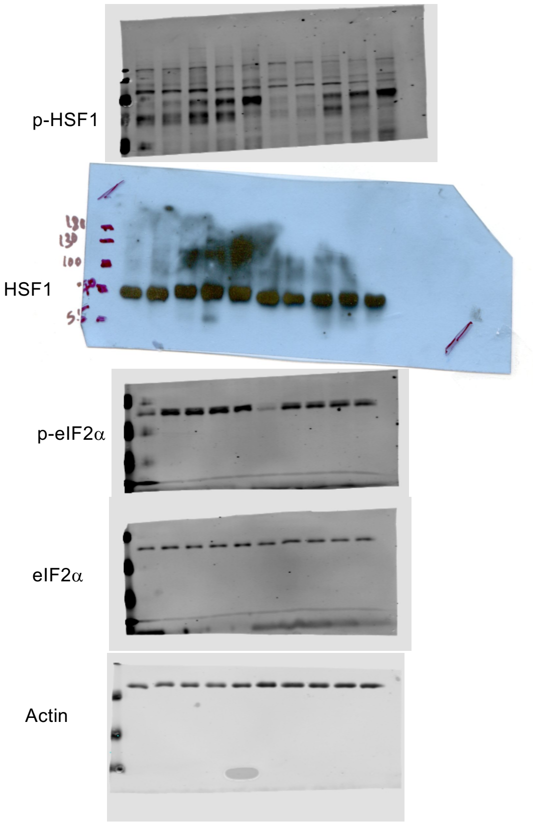

Original data figure 5B

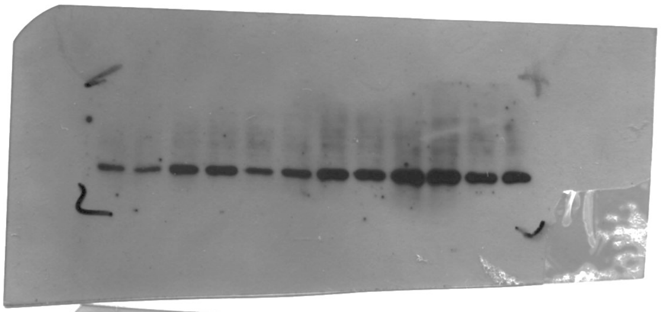

Ⓟ eIF2α

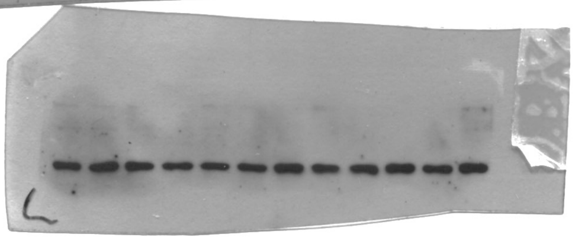

eIF2α

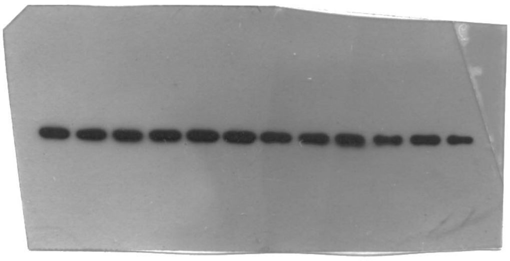

Actin

Original data figure 6B

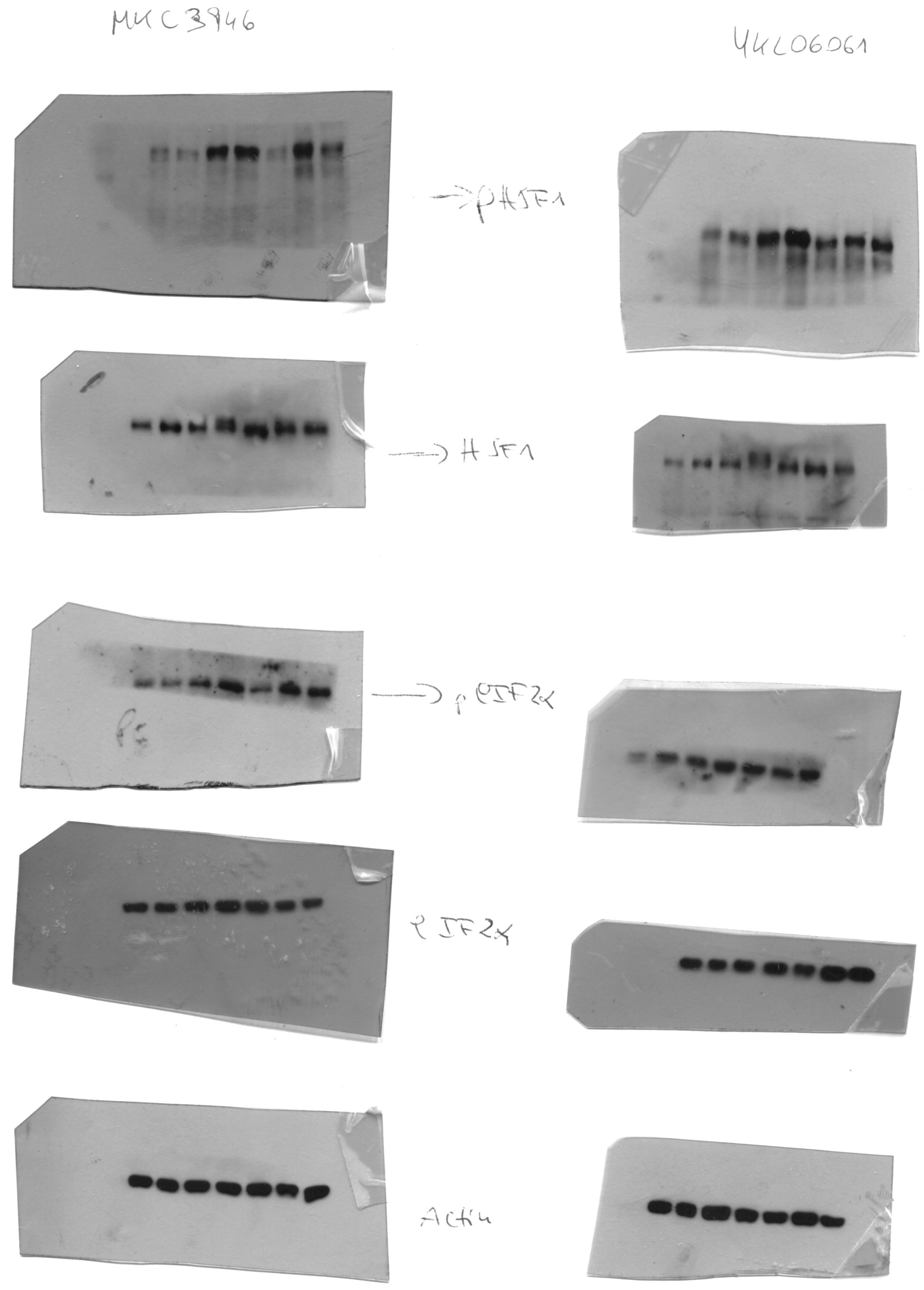

Supplement: Supplementary file 9 — Original Data File [file 41420_2022_1202_MOESM9_ESM.pdf]
