# Supplementary material for: Proteotoxic stress-induced apoptosis in cancer cells: understanding the susceptibility and enhancing the potency
Source: Cell Death Discov. 2022 Oct 4;8:407. doi: 10.1038/s41420-022-01202-2 (PMC9531228; doi:10.1038/s41420-022-01202-2)
Supplement: Supplementary file 1 — Authorship change agreement [file 41420_2022_1202_MOESM1_ESM.pdf]

To the Editor
CDDISCOVERY

REF Manuscript Number: CDDISCOVERY-22-4683R1

Title: Proteotoxic stress-induced apoptosis in cancer cells: understanding the susceptibility and enhancing the potency

Authors: Claudio Brancolini, Luca Iuliano, Emiliano Dalla, Raffaella Picco, Showmeya Mallavarapu, Martina Minisini, and Eleonora Malavasi

Dear editors

Here are the requested consents of all authors for the inclusion of the new author Eleonora Malavasi.

With the best regards

Claudio Brancolini

Re: CDDISCOVERY-22-4683R1 Initial Quality Check – Inbox

Luca Iuliano
Re: CDDISCOVERY-22-4683R1 Initial Quality Check
To: Raffaella Picco, Sowmya Mallavarapu, Claudio Brancolini, Cc: Emiliano Dalla, MARTINA MINISINI

Inbox - Exchange 16:36
Details

Dear Prof. Brancolini, dear Editorial Office,

I, Luca Iuliano, co-author of the manuscript entitled Proteotoxic stress-induced apoptosis in cancer cells: understanding the susceptibility and enhancing the potency (CDDISCOVERY-22-4683R), hereby declare that I have read and I agree to the changes introduced to the co-authors list of the manuscript, which is now as follow:

Luca Iuliano, Emiliano Dalla, Raffaella Picco, Showmeya Mallavarapu, Martina Minisini, Eleonora Malavasi and Claudio Brancolini

Sincerely,
Luca Iuliano

Emiliano Dalla
R: CDDISCOVERY-22-4683R1 Initial Quality Check
To: Claudio Brancolini, Luca Iuliano, MARTINA MINISINI, Sowmya Mallavarapu

Inbox - Exchange 14:18

Dear Prof. Brancolini, dear Editorial Office,

I, the undersigned, hereby declare that I have read and that I agree to the changes introduced to the co-authors list of manuscript CDDISCOVERY-22-4683R (entitled **Proteotoxic stress-induced apoptosis in cancer cells: understanding the susceptibility and enhancing the potency**), which is now as follows:

Luca Iuliano, Emiliano Dalla, Raffaella Picco, Showmeya Mallavarapu, Martina Minisini, Eleonora Malavasi and Claudio Brancolini

Sincerely,
Emiliano Dalla.

Da: Claudio Brancolini <claudio.brancolini@uniud.it>
Inviato: venerdì 23 settembre 2022 14:10
A: Emiliano Dalla <emiliano.dalla@uniud.it>; Iuliano Luca <iuliano.luca@spes.uniud.it>; Martina Minisini <minisini.martina@spes.uniud.it>; sowmyamallavarapu7 <sowmyamallavarapu7@gmail.com>
Oggetto: Fwd: CDDISCOVERY-22-4683R1 Initial Quality Check

Dear co-authors

As you can read from the editor's email below, you have to agree the change in the co-authors and accept the new co-author Eleonora Malavasi.

The new list of co-authors of the manuscript CDDISCOVERY-22-4683R entitled **Proteotoxic stress-induced apoptosis in cancer cells: understanding the susceptibility and enhancing the potency**
and their order is the following: Luca Iuliano, Emiliano Dalla, Raffaella Picco, Showmeya Mallavarapu, Martina Minisini, Eleonora Malavasi and Claudio Brancolini
see the instruction below, essentily you have to reply to this email

can you do it asap

Re: CDDISCOVERY-22-4683R1 Initial Quality Check – Inbox

RP **Raffaella Picco** Inbox - Exchange 15:55

Re: CDDISCOVERY-22-4683R1 Initial Quality Check

To: Sowmya Mallavarapu, Claudio Brancolini, Cc: Emiliano Dalla, Luca Iuliano, MARTINA MINISINI

Details

Dear Prof. Brancolini, dear Editorial Office,

I, Raffaella Picco, co-author of the manuscript entitled **Proteotoxic stress-induced apoptosis in cancer cells: understanding the susceptibility and enhancing the potency** (CDDISCOVERY-22-4683R), hereby declare that I have read and I agree to the changes introduced to the co-authors list of the manuscript, which is now as follow:

Luca Iuliano, Emiliano Dalla, Raffaella Picco, Showmeya Mallavarapu, Martina Minisini, Eleonora Malavasi and Claudio Brancolini

Raffaella Picco

Ottenere Outlook per Android

From: sowmya mallavarapu <sowmyamallavarapu7@gmail.com>

SM **Sowmya Mallavarapu** Inbox - Exchange 15:49

Re: CDDISCOVERY-22-4683R1 Initial Quality Check

To: Claudio Brancolini, Cc: Emiliano Dalla, Luca Iuliano, Raffaella Picco, MARTINA MINISINI

Details

Dear Prof. Brancolini, dear Editorial Office,

I, the undersigned, hereby declare that I have read and that I agree to the changes introduced to the co-authors list of manuscript CDDISCOVERY-22-4683R (entitled **Proteotoxic stress-induced apoptosis in cancer cells: understanding the susceptibility and enhancing the potency**), which is now as follows:

Luca Iuliano, Emiliano Dalla, Raffaella Picco, Showmeya Mallavarapu, Martina Minisini, Eleonora Malavasi and Claudio Brancolini

Sincerely,
Showmeya

MM **MARTINA MINISINI** Inbox - Exchange 15:18

Re: CDDISCOVERY-22-4683R1 Initial Quality Check

To: Claudio Brancolini, Emiliano Dalla, Cc: Luca Iuliano, Sowmya Mallavarapu, Raffaella Picco

Details

Dear Prof. Brancolini, dear Editorial Office,

I, Martina Minisini, co-author of the manuscript entitled **Proteotoxic stress-induced apoptosis in cancer cells: understanding the susceptibility and enhancing the potency** (CDDISCOVERY-22-4683R), hereby declare that I have read and I agree to the changes introduced to the co-authors list of the manuscript, which is now as follow:

Luca Iuliano, Emiliano Dalla, Raffaella Picco, Showmeya Mallavarapu, Martina Minisini, Eleonora Malavasi and Claudio Brancolini

Martina Minisini

From: Claudio Brancolini <claudio.brancolini@uniud.it>
